# Primary Pediatric Brain Tumors in a Tertiary Referral Center in Iran: A 13-Year Retrospective Analysis

**DOI:** 10.34172/aim.34594

**Published:** 2025-10-01

**Authors:** Ghazaleh Kheiri, Sadra Kheiri, Milad Shafizadeh, Keyvan Tayebi Meybodi, Farideh Nejat, Zohreh Habibi

**Affiliations:** ^1^Department of Neurosurgery, Children’s Medical Center, Tehran University of Medical Sciences, Tehran, Iran; ^2^Department of Neurosurgery, Isfahan University of Medical Sciences, Isfahan, Iran; ^3^Department of Neurosurgery, Shariati Hospital, Tehran University of Medical Sciences, Tehran, Iran

**Keywords:** Central nervous system, Pediatric brain tumor, Tumor epidemiology

## Abstract

**Background:**

Pediatric central nervous system (CNS) tumors are among the most common childhood malignancies and a leading cause of cancer-related death. This study aimed to describe the histopathological spectrum of primary brain tumors in children over a 13-year period in a tertiary referral center in Iran.

**Methods:**

In this existing data study (EDS), we reviewed the medical records of 549 patients aged 0–17 years who underwent surgical resection of brain tumors between 2008 and 2020. Tumor characteristics, including histology, location, and World Health Organization (WHO 2016) grade, along with patient age and sex, were extracted. Descriptive and comparative analyses were conducted. Temporal trends for single-center in the number of pediatric brain tumor surgeries performed in our center were assessed using Poisson regression.

**Results:**

The mean age at diagnosis was 5.1 years, with a male-to-female ratio of 1.3:1. Pilocytic astrocytoma (20.9%) was the most common tumor, followed by medulloblastoma (15.3%) and ependymoma (11.3%). Tumors were nearly evenly distributed between supratentorial (48.6%) and infratentorial (48.5%) regions. Grade I tumors were most frequent overall, while Grade IV tumors were significantly more common in the infratentorial space (*P*<0.001). No significant differences in tumor distribution were observed by sex or age group. The number of surgeries increased significantly over time (incidence rate ratio: 1.127/year, *P*<0.001), with no subgroup differences in temporal trend.

**Conclusion:**

This single-center study provides institutional-level insights into the histopathological characteristics of pediatric brain tumors. The predominance of low-grade tumors observed emphasizes the need for national pediatric neuro-oncology registries, particularly in low- and middle-income countries, to enable more representative data collection and planning.

## Introduction

 Brain and central nervous system (CNS) tumors are a diverse group of neoplasms that originate from various structures within the CNS. According to Global Cancer Statistics, about 308,000 new cases of brain and CNS cancers were diagnosed worldwide in 2020, leading to over 251,000 deaths.^1^ In children, CNS tumors are the second most common type of cancer and one of the leading causes of cancer-related deaths.^2^ These tumors vary widely in their biology and behavior, and importantly, pediatric CNS tumors differ significantly from those seen in adults in terms of incidence, histological types, and outcomes.^3^

 Data from the Global Burden of Disease (GBD) 2021 study indicate that CNS cancers continue to have high incidence and mortality rates worldwide, with a steady increase observed from 1990 to 2021. Notably, the rise in incidence varies considerably across regions, which may reflect differences in diagnostic capacity, access to treatment, and potentially unidentified environmental, genetic, or other risk factors.^4^

 The incidence patterns of pediatric CNS tumors also vary geographically. Studies from Canada and France have shown stable trends, while data from the United States suggest a slight but statistically significant rise in incidence rates.^5-7^ According to the 2023 CBTRUS Statistical Report, the average annual incidence rate of primary CNS tumors in individuals aged 0–19 years in the United States was 5.92 per 100,000 during the period 2016–2020.^8^ Globally, the rate is estimated at 2.8 per 100,000, while in Iran, it is reported to be lower, around 1.43 per 100,000.^2,9^ A previous Iranian study examining data from 1996 to 2013 found a rising trend in pediatric CNS tumor cases during that period.^10^

 Epidemiological data also show variation by age and sex. CNS tumors tend to be more common in males, and the highest incidence is usually seen in children under the age of five. In this age group, malignant tumors are especially frequent.^9,10^ These patterns emphasize the importance of ongoing surveillance and research, particularly in younger children.

 One of the challenges in tracking incidence trends over time is the evolving classification of CNS tumors. Updates to diagnostic criteria, especially the introduction of molecular markers in the 2016 WHO classification, have improved accuracy but also made long-term comparisons more complex.^11^

 Despite global data, regional reports from low- and middle-income countries remain limited. The Iranian national cancer registry provides baseline estimates, but detailed information is lacking regarding histological subtypes, tumor grade, location, and evolving classification schemes. Moreover, long-term institutional data capturing these characteristics in a consistent, WHO-aligned manner are scarce. This study presents a 13-year retrospective analysis of primary pediatric brain tumors in Children’s Medical Center, a major tertiary referral hospital in Tehran, Iran. Using the 2016 WHO classification, we describe tumor distribution by histology, location, grade, and patient demographics. While not population-based, this analysis offers valuable insights into patterns observed in a large pediatric neuro-oncology center and highlights the need for improved diagnostic infrastructure and centralized data collection in Iran.

## Materials and Methods

###  Study Design

 We conducted an existing data study (EDS) of pediatric brain tumors treated in a major tertiary referral center. The study included all children (aged 0–17 years) who underwent neurosurgical intervention with histopathological confirmation of a primary intracranial tumor in Children’s Medical Center in Tehran, Iran, between March 2008 and March 2020. Spinal cord tumors and secondary (metastatic) brain lesions were excluded. The study adhered to the STROBE (Strengthening the Reporting of Observational Studies in Epidemiology) guidelines for reporting observational research.

###  Data Collection and Variables

 Data were extracted from medical records and included patient age at diagnosis, sex, tumor histology, anatomical location, and World Health Organization (WHO 2016) tumor grade. Only the first neurosurgical event for each patient was analyzed to avoid duplication. Cases involving vascular malformations, skull and dermal lesions, non-neoplastic cysts, or metastatic tumors, and spinal cord tumors were excluded. For age-based subgroup analysis, patients were categorized as infants (0–1 year), toddlers/preschool-aged (1–5 years), and school-aged children/adolescents (5–17 years).

###  Statistical Analysis

 Categorical variables were summarized using frequencies and percentages, and continuous variables were presented as means ± standard deviation (SD). Group comparisons of continuous variables were conducted using independent-samples t-tests or one-way analysis of variance (ANOVA), as appropriate. Chi-square tests were applied for comparisons of categorical variables. Trends over time in the number of pediatric brain tumor cases managed in our center were evaluated using Poisson regression modeling. All tests were two-tailed, and p-values less than 0.05 were considered statistically significant. Analyses and visualizations were performed using IBM® SPSS® Statistics, Version 25.

## Results

###  Patient Characteristics

 A total of 549 pediatric brain tumor cases were included in the study. The mean age at diagnosis was 5.08 years, ranging from 2.5 months to 17 years. Males accounted for 56.5% (310 cases), and females for 43.5% (239 cases), resulting in a male-to-female ratio of 1.3:1. There was no significant association between age at diagnosis and gender (*P* = 0.355) (Table 1).

**Table 1:** Patient Characteristics

**Variable**	**Category**	**n (%) or Value**
Total patients		549
Age		Mean: 5.08 years (2 months–17 years)
Sex	Male	310 (56.5%)
	Female	239 (43.5%)
Tumor grade	Grade I	219 (39.9%)
	Grade II	110 (20.0%)
	Grade III	51 (9.3%)
	Grade IV	163 (29.7%)
Tumor location	Supratentorial	267 (48.6%)
	Infratentorial	266 (48.5%)
	Other	16 (2.9%)

###  Tumor Histology

**Figure 1:**
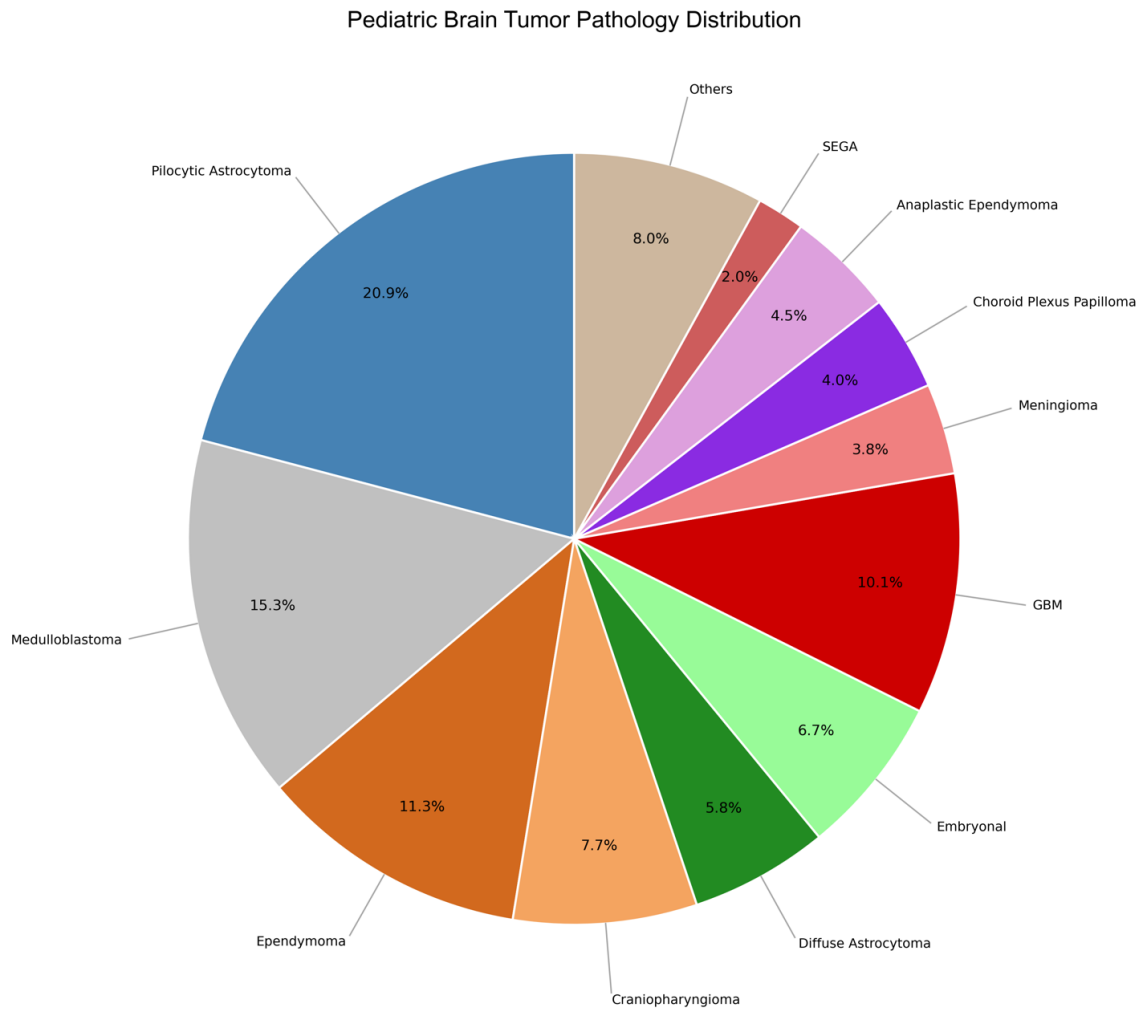
Pediatric Brain Tumor Histology Distribution (2008-2020). The chart displays the relative frequencies of histopathological tumor types among 549 pediatric patients (aged 0–17 years) who underwent brain tumor surgery. Pilocytic astrocytoma was the most common tumor (22.8%), followed by medulloblastoma (16.6%) and ependymoma (12.3%). Percentages indicate each tumor’s proportion within the total surgical cohort

 Pilocytic astrocytoma was the most common histopathological subtype, accounting for 20.9% of all cases, followed by medulloblastoma (15.3%), ependymoma (11.3%), and craniopharyngioma (7.7%). Age-stratified histological patterns showed pilocytic astrocytoma as the most frequent tumor across all age groups. In infants (0–1 years), choroid plexus papilloma and ependymoma were also prevalent. In the 1–5-year group, medulloblastoma and craniopharyngioma followed pilocytic astrocytoma. Among patients aged 5–17 years, pilocytic astrocytoma and medulloblastoma were most common (Table 2, Figure 1). Frequencies reflect relative proportions within each age category.

**Table 2:** Most common histopathology in different age groups

**Age group**	**Most common pathology**
0-1	Pilocytic astrocytoma (19%) Choroid plexus papilloma (12%) Ependymoma (10%) Medulloblastoma (9%) Anaplastic ependymoma (7%) ATRT (7%) Embryonal (7%)
1-5	Pilocytic astrocytoma (22.8%) Medulloblastoma (18.3%) Ependymoma (15.6%) Embryonal (8.5%) Craniopharyngioma (8%) Diffuse astrocytoma (6.7%)
5-17	Pilocytic astrocytoma (19.1%) Medulloblastoma (15.7%) Craniopharyngioma (9.8%) Ependymoma (7.8%) GBM (7.8%)

ATRT: Atypical teratoid rhabdoid tumor; GBM: Glioblastoma multiforme.

###  Tumor Grade and Sex Distribution

**Figure 2:**
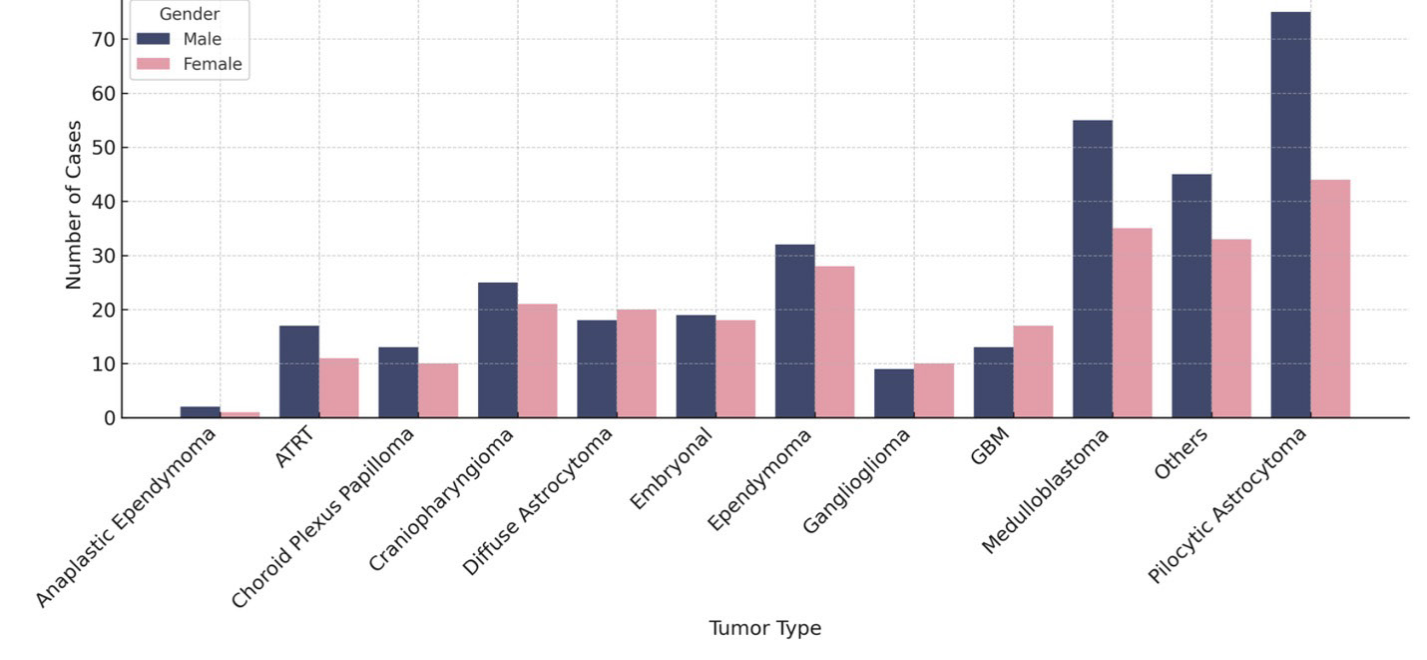
Distribution of pediatric Brain Tumors by Gender and Histology

 Among all tumors, 219 (39.9%) were WHO Grade I, 110 (20.0%) Grade II, 51 (9.3%) Grade III, and 163 (29.7%) Grade IV. Grade I tumors were more frequent in both sexes, though some tumor subtypes (e.g. schwannoma, pilomyxoid astrocytoma, oligodendroglioma, lymphoma, GBM, ganglioglioma, diffuse astrocytoma, anaplastic astrocytoma) were more common in females (Figure 2). No significant association was found between tumor grade and gender (*P* = 0.575).

###  Tumor Location and Grade

**Figure 3:**
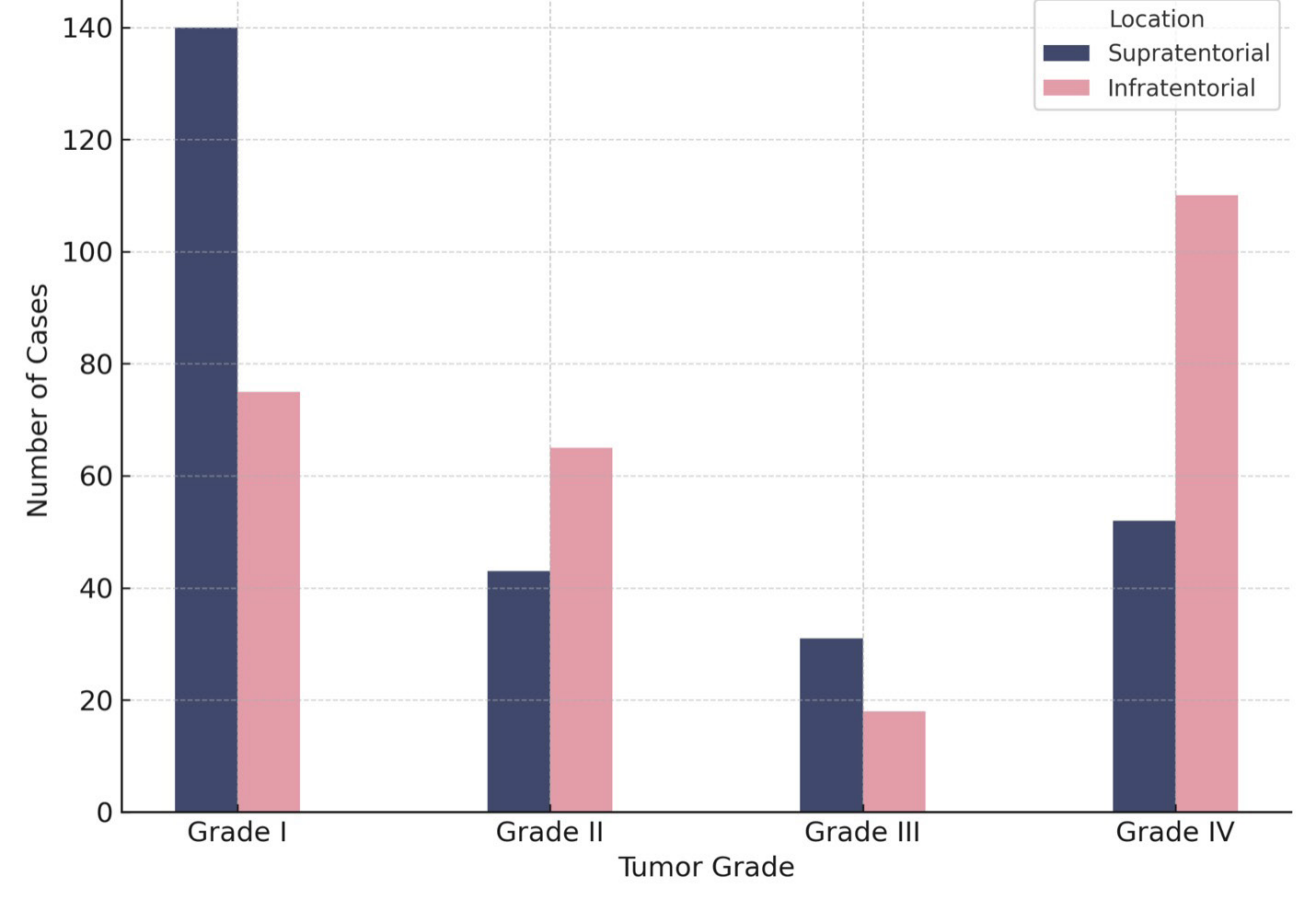
Tumor Grade Distribution by Location

 Tumors were nearly equally distributed between supratentorial (48.6%) and infratentorial (48.5%) compartments, with a small proportion (2.9%) classified as “other” (Table 2). Grade I tumors predominated in the supratentorial region (52.5%), while Grade IV tumors were the most frequent infratentorially (40.9%). A significant association was observed between tumor grade and location (*P* < 0.001), with high-grade tumors more often located in the posterior fossa (Figure 3). The mean age was similar across tumor grades: 5.2 years (Grade I), 4.8 (Grade II), 5.0 (Grade III), and 5.1 (Grade IV), with no significant difference by grade (*P* = 0.819).

###  Annual Case Volume Trend

**Figure 4:**
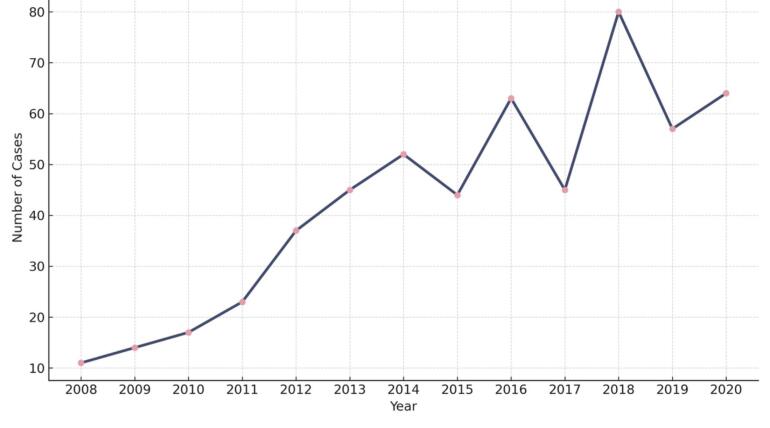
Annual Number of Pediatric Brain Tumor Cases (2008-2020)

 The number of pediatric brain tumor cases managed surgically in our center increased significantly over the 13-year period. Poisson regression revealed a 1.127-fold increase in the annual number of cases treated (*P* < 0.001), suggesting a rising referral volume or improved diagnostic access (Figure 4). This trend represents center-based surgical case counts and does not imply population-level incidence.

###  Subgroup Temporal Patterns

**Figure 5:**
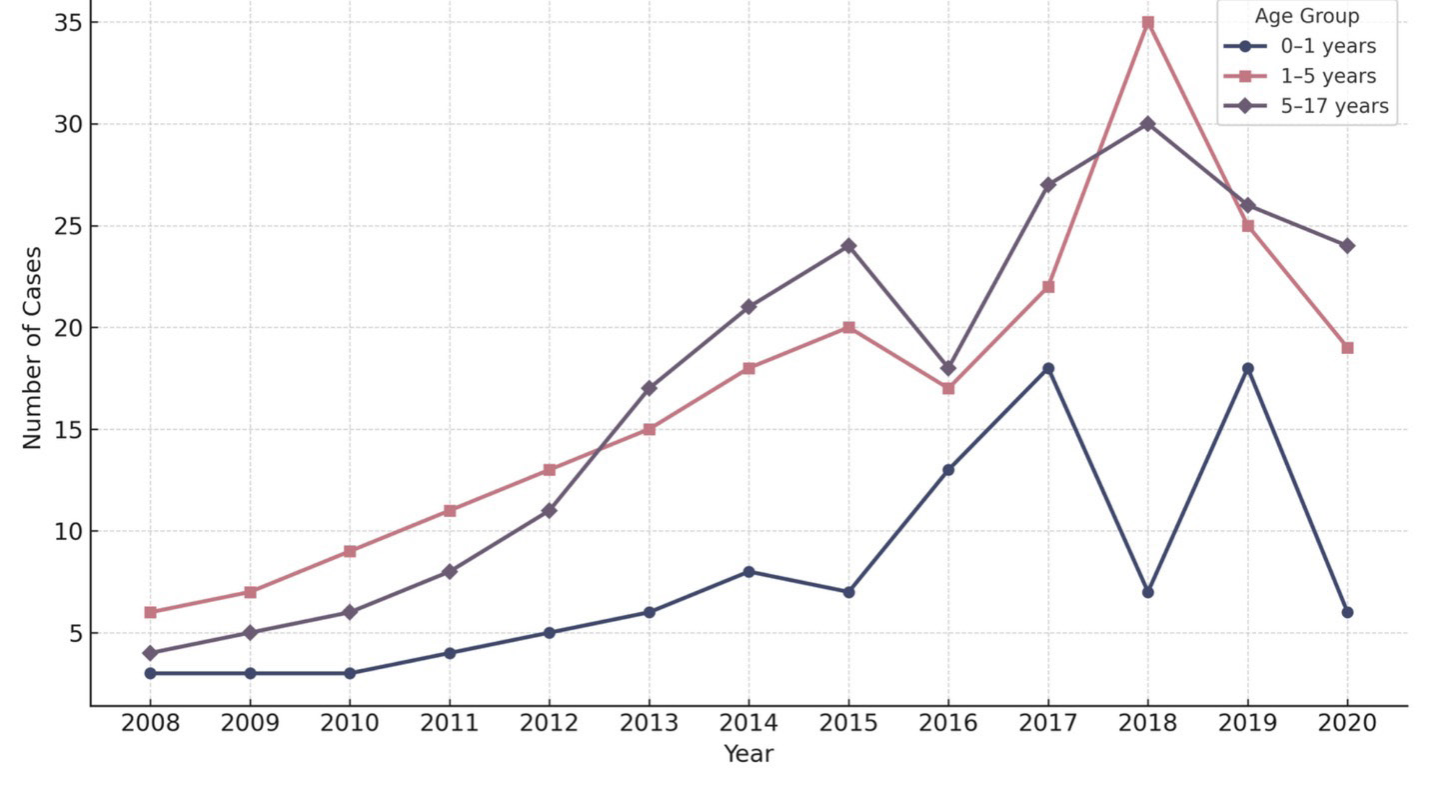
Annual Incidence of Pediatric Brain Tumors by Age Group (2008-2020)

 Trends in the number of cases over time were examined across clinical subgroups. No significant differences were observed in the annual increase of cases by tumor location (supratentorial vs. infratentorial, *P* = 0.848), sex (*P* = 0.411), tumor grade (low- vs. high-grade, *P* = 0.525), or age group (*P* = 0.232) (Figure 5).

## Discussion

 In this study, we reviewed the clinical and histopathological features of 549 children diagnosed with brain tumors who underwent surgery in our center. The average age at diagnosis was 5.08 years, ranging from just a couple of months old to 17 years. This is in line with a previous report from Iran that found a mean age of 5.5 years.^10^ Compared to earlier studies from Africa, South Korea, and other regions of Iran,^12-14^ our patient group tended to be slightly younger. This might be due to the fact that our center is a major referral hospital for younger children, which could explain the age distribution we observed.

 Previous studies have shown that pediatric CNS tumors are generally more common in males than females.^7,15,16^ Our findings were consistent with this pattern, with a male-to-female ratio of 1.3:1 across the entire cohort. However, a closer analysis of specific tumor types revealed that several were more frequently observed in females. These included schwannoma, pilomyxoid astrocytoma, oligodendroglioma, brain lymphoma, GBM, ganglioglioma, diffuse astrocytoma, and anaplastic astrocytoma, all with an M/F ratio below 1. A similar pattern was reported in a previous study of 542 pediatric brain tumor cases, where female predominance was observed in tumors such as ependymoma, craniopharyngioma, meningioma, diffuse astrocytoma, anaplastic ependymoma, germinoma, and choroid plexus tumors.^17^ These findings suggest that while a general male predominance is observed, certain tumor subtypes may show a different sex distribution.

 Astrocytomas, embryonal tumors, and malignant gliomas have been reported as the most frequent CNS tumors in children.^18^ In our cohort, pilocytic astrocytoma was the most common histopathological type, accounting for 20.9% of all cases. This was followed by medulloblastoma (15.3%), ependymoma (11.3%), and craniopharyngioma (7.7%). These findings are in line with several large epidemiological studies,^17,19,20^ and closely resemble the results of another Iranian study, which also identified astrocytoma as the most frequent pediatric brain tumor, followed by medulloblastoma, ependymoma, craniopharyngioma, and meningioma.^12^

 In most previously published series, astrocytomas consistently rank as the most common pediatric brain tumor.^18^ However, some variation does exist. For example, Aghayan et al^10^ reported medulloblastoma as the most prevalent tumor type, followed by ependymoma (12.4%) and pilocytic astrocytoma (10.9%). Similarly, a population-based study from the French National Registry found that approximately 57% of pediatric brain tumors were gliomas, with pilocytic astrocytoma representing the majority.^7^

 The most commonly reported tumors in the posterior fossa include medulloblastoma, cerebellar astrocytoma, brainstem glioma, and ependymoma, whereas supratentorial tumors are more often other astrocytomas and glioneuronal tumors.^21^ In our study, we observed an almost equal distribution of tumors between these two regions, with a supratentorial-to-infratentorial (S/I) ratio of 1:1. This mirrors the findings of Alexiou et al,^19^ who also reported a balanced distribution across age groups, although they noted a slightly higher prevalence of posterior fossa tumors in children aged 1–2 years.

 Globally, about 55% of pediatric brain tumors are located in the infratentorial space, with the remaining 45% found in the supratentorial space.^22^ However, the anatomical distribution of tumors varies across studies and regions. For instance, researchers from China, France, and Brazil have reported a predominance of supratentorial tumors,^23-25^ while studies from Iran, Pakistan, India, and Canada have observed a higher proportion of infratentorial tumors.^9,26,27^ It is also possible that infratentorial tumors are underestimated in some settings, particularly due to the non-biopsied nature of lesions like diffuse intrinsic pontine glioma (DIPG).

 Previous studies have shown that posterior fossa tumors tend to occur more frequently in children aged 1 to 11 years, while infants under one year and older children are more likely to develop supratentorial tumors.^28,29^ In our cohort, the most frequently observed tumor grade in both males and females was Grade I, and there was no significant association between tumor grade and sex.

 In our study, the most common tumor grade in the supratentorial region was Grade I, accounting for 52.5% of cases, whereas Grade IV tumors were most frequent in the infratentorial space, making up 40.9%. This difference was statistically significant, indicating a strong association between tumor grade and anatomical location. Among infratentorial tumors, the most frequently observed histopathologies were medulloblastoma (31.6%), followed by ependymoma (23.7%) and pilocytic astrocytoma (23.7%). In contrast, the most common supratentorial tumors were pilocytic astrocytoma (18%), craniopharyngioma (15%), and embryonal tumors (8.6%).

 We also found that pilocytic astrocytoma was the most frequent histological type across all age groups, highlighting its predominance in the pediatric population. This observation aligns with previous literature, although age-specific tumor distribution patterns can vary. For example, Zhou et al^25^ reported that malignant and infratentorial tumors were most prevalent in children aged 0–2 years. Similarly, in our study, a large proportion of the tumors diagnosed in patients aged 0–1 year were malignant, suggesting a higher burden of aggressive histologies in early infancy.

 A systematic review and meta-analysis estimated the global incidence rate of all primary brain tumors to be 10.82 per 100,000 individuals per year (95% CI: 8.63–13.56).^30^ A population-based study using data from the Italian Cancer Registry found that the overall incidence of brain tumors increased from 1985 to 2005, primarily due to a rise in benign tumors, while the rate of malignant tumors remained stable.^31^ Similarly, the GBD 2019 study reported a 94.35% increase in the global prevalence of CNS tumors over the period 1990–2019, indicating a growing global burden.^32^

 While our study is based on a single tertiary referral center and does not represent population-level surveillance, we analyzed temporal trends in the number of pediatric brain tumor cases managed surgically between 2008 and 2020. The data revealed a statistically significant annual increase of 1.127 times in the number of cases. This observed rise may reflect improved diagnostic access, greater referral volume, or expanded neurosurgical services in our center over time, rather than a true increase in incidence in the general population.These findings are consistent with prior reports, including a study by Aghayan et al,^10^ which showed a rising incidence of pediatric brain tumors in Iran between 1996 and 2013. Other studies from the United States and Europe have also documented increasing trends from the 1970s to the 1990s, largely attributed to advances in diagnostic imaging, particularly the widespread adoption of MRI.^33-35^ A study from Finland reported a more modest annual increase of 0.8% in pediatric CNS tumors between 1990 and 2017, without significant changes in tumor location or histopathology.^36^ In contrast, a French registry-based study covering 2000 to 2008 did not find any significant trend in CNS tumor incidence during that period.^7^

 Although our results should be interpreted with caution due to the referral-based nature of our dataset, they offer insight into institutional patterns and contribute valuable local data to the limited literature on pediatric brain tumors in Iran.

 This study has several limitations that should be considered. First, it was conducted in a single tertiary referral center, which may introduce referral bias and limit the generalizability of the findings to the broader pediatric population in Iran. As a referral hospital, our center may receive more complex or advanced cases, which could influence the distribution of tumor types and grades. Second, because this was a retrospective review, the completeness and consistency of medical records may have affected data accuracy, particularly for variables like tumor location, imaging findings, and long-term outcomes. Third, intra- versus extra-axial tumor location was not consistently documented in the medical records and could not be reliably included in the analysis. Fourth, our trend analysis reflects temporal patterns in the number of cases managed in our center, rather than population-based incidence estimates. As such, the findings should not be interpreted as national trends. Additionally, we only included patients who underwent surgery with histopathological confirmation, which may exclude tumors like DIPG, which are typically diagnosed radiologically and not biopsied.

## Conclusion

 This 13-year retrospective study provides an updated overview of the histopathological and demographic characteristics of pediatric brain tumors treated in a major referral center in Iran. Pilocytic astrocytoma was the most common tumor across all age groups, with medulloblastoma and ependymoma also frequently observed. Tumor grade and anatomical location were significantly associated, with high-grade tumors more often located in the infratentorial space.

 While the number of surgically managed cases increased over time, this trend likely reflects changes in referral patterns and institutional capacity rather than true population-level incidence. These findings contribute to the limited data on pediatric neuro-oncology in Iran and highlight the need for nationwide cancer registries and multicenter collaboration to support surveillance, early diagnosis, and evidence-based care.
